# Molecular Epidemiology of *Escherichia coli* with Resistance against Third-Generation Cephalosporines Isolated from Deployed German Soldiers—A Retrospective Assessment after Deployments to the African Sahel Region and Other Sites between 2007 and 2016

**DOI:** 10.3390/microorganisms10122448

**Published:** 2022-12-11

**Authors:** Frederik Pankok, Frieder Fuchs, Ulrike Loderstädt, Martin Kaase, Carsten Balczun, Simone Scheithauer, Hagen Frickmann, Ralf Matthias Hagen

**Affiliations:** 1 Institute for Infection Control and Infectious Diseases, University Medical Center Göttingen, 37075 Göttingen, Germany; 2 Department of Microbiology and Hospital Hygiene, Bundeswehr Central Hospital Koblenz, 56070 Koblenz, Germany; 3 Institute for Medical Microbiology, Immunology and Hygiene, University of Cologne, Medical Faculty and University Hospital of Cologne, 50931 Cologne, Germany; 4 Department of Microbiology and Hospital Hygiene, Bundeswehr Hospital Hamburg, 20359 Hamburg, Germany; 5 Institute for Medical Microbiology, Virology and Hospital Hygiene, University Medicine Rostock, 18057 Rostock, Germany

**Keywords:** cephalosporine resistance, Enterobacterales, *Escherichia coli*, antibiotic resistance, surveillance, sub-Saharan Africa, Sahel, soldiers, Mali, Djibouti, Sudan, South Sudan, ESBL

## Abstract

Colonization and infection with bacteria with acquired antibiotic resistance are among the risks for soldiers on international deployments. Enterobacterales with resistance against third-generation cephalosporines are amongst the most frequently imported microorganisms. To contribute to the scarcely available epidemiological knowledge on deployment-associated resistance migration, we assessed the molecular epidemiology of third-generation cephalosporine-resistant *Escherichia coli* isolated between 2007 and 2016 from German soldiers after deployments, with a particular focus on the African Sahel region. A total of 51 third-generation cephalosporine-resistant *E. coli* isolated from 51 military returnees from deployment collected during the assessment period between 2007 and 2016 were subjected to short-read next-generation sequencing analysis. Returnees from the Sahel region (Djibouti, Mali, South Sudan, Sudan, Sudan, and Uganda) comprised a proportion of 52.9% (27/51). Repeatedly isolated sequence types according to the Warwick University scheme from returnees from the Sahel region were ST38, ST131, and ST648, confirming previous epidemiological assessments from various sub-Saharan African regions. Locally prevalent resistance genes in isolates from returnees from the Sahel region associated with third-generation resistance were *bla*_CTX-M-15_, *bla*_CTX-M-27_, *bla*_CTX-M-1_, *bla*_TEM-169_, *bla*_CTX-M-14_, *bla*_CTX-M-99_-like, *bla*_CTX-M-125_, *bla*_SHV-12_, and *bla*_DHA-1_, while virulence genes were *east1*, *sat*, and *tsh* in declining order of frequency of occurrence each. In line with phenotypically observed high resistance rates for aminoglycosides and trimethoprim/sulfamethoxazole, multiple associated resistance genes were observed. A similar, slightly more diverse situation was recorded for the other deployment sites. In summary, this assessment provides first next-generation sequencing-based epidemiological data on third-generation cephalosporine-resistant *E. coli* imported by deployed German soldiers with a particular focus on deployments to the Sahel region, thus serving as a small sentinel. The detected sequence types are well in line with the results from previous epidemiological assessments in sub-Saharan Africa.

## 1. Introduction

Soldiers are at increased risk of acquiring multidrug-resistant bacteria in the course of military deployments to war and crisis zones [1,2]. As demonstrated recently [3,4], anthropological and socioeconomic factors associated with poor governance are stronger predictors of antimicrobial resistance spread than nonadherence to good clinical practice regarding the prescription of antibiotic drugs. Climatic influences and staffing of medical facilities have also been suggested to play a role [5,6]. In particular, antimicrobial-resistant Gram-negative bacteria are frequently imported from deployments in war and crisis settings [7] with a quantitative dominance of Enterobacterales with resistance against third-generation cephalosporines. As shown in a recent screening assessment, German deployed soldiers are no exemption from this rule [8].

The majority of Enterobacterales with resistance against third-generation cephalosporines from a previous study [8] were imported from African deployment sites with particular emphasis on the Sahel region, an area where German soldiers have been deployed on European Union and United Nations missions. This observation is not surprising, as resistant Enterobacterales are common in sub-Saharan Africa [9], especially in settings where resistance surveillance activity is scarce and antibiotic therapy is not guided by diagnostic approaches or infectious disease experts [10].

From the Sahel region, a sub-Saharan habitat associated with particularly unfavorable climatic and living conditions [11], cephalosporine-resistant *Escherichia coli* were isolated from German soldiers after deployments to Mali, Sudan, South Sudan, and Djibouti [8]. Among the international literature focusing on those areas, most resistance surveillance data are available for the Sudanese region including South Sudan and neighboring Uganda. In Sudan and South Sudan, third-generation resistance rates of partly more than 30% have been reported for Enterobacterales infecting or colonizing the local population [12,13,14,15,16,17,18,19,20,21] including enteric and typhoid salmonellae in human or animal hosts [22,23,24]. First hints for increasing local resistance rates have been known for decades [25,26,27,28] with the exception of some remote, isolated areas of the country [29]. Most recently, carbapenem resistance was identified in *Klebsiella* spp., which show multiclonal circulation in Sudanese hospitals [30,31], as well as in *E. coli* in Sudanese water sources [32]. Resistant Enterobacterales in Sudan and South Sudan have also been isolated from environmental sources such as farm animals [33] and even from bank notes [34]. Over-the-counter availability of antibiotic drugs [35] and high rates of nosocomial hospital infections [36] also contribute to the local resistance spread. Although first reports on transmissible genetic elements conferring antibiotic resistance in Sudan and South Sudan were already published in the 1980s and 1990s [37,38], the available literature on their local molecular epidemiology is still scarce [39,40]. In neighboring Uganda, plasmid-mediated ampC beta-lactamase (pAmpC)-encoding genes are frequent causes of third-generation cephalosporine resistance [41].

From Western African Mali, severe systemic infections due to extended-spectrum beta-lactamase (ESBL-)positive Enterobacterales have been reported [42,43]. Approximately two-thirds of the Enterobacterales isolated from blood cultures were resistant against third-generation cephalosporines in previous assessments [44,45,46]. While third-generation cephalosporine-resistant Enterobacterales usually spread as colonizing bacterial flora [47,48,49], more severe infections have been reported from immunocompromised individuals [50]. Molecular assessments of resistant strains from Mali are nevertheless scarcely available. In European soldiers deployed to Mali in 2013 and 2014, the enteric carriage of Enterobacterales expressing extended spectrum beta lactamases (ESBL) was reported from about one out of three assessed soldiers with diarrhea [51,52], potentially associated with transmission from food sources [53].

In Djibouti, resistance in Enterobacterales drastically increased from the abundance of beta-lactamases in the 1980s and 1990s [54,55,56] to ceftazidime susceptibility of less than 5% in a recent assessment associated with ESBL enzymes [57]. Interestingly, short therapy courses of infectious diarrhea with non-beta-lactam antibiotics such as azithromycin, levofloxacin, or rifaximin still show reliable therapeutic effects in this country [58]; 1 day therapy schemes do not even seem to be associated with considerable resistance selection [59].

Among other countries of the Sahel region, high rates of cephalosporine resistance have also been reported from Senegal in patients and livestock [60,61,62,63,64,65], as well as from Niger [66,67], Burkina Faso [68], and Chad [69], with combined ESBL- and AmpC-mediated resistance in strains from human patients [60] and carbapenem-resistant *Klebsiella* spp., even from wild animals [70] in Senegal.

To provide new pieces to the puzzle of the global molecular epidemiology of resistant bacterial clones, third-generation cephalosporine-resistant *Escherichia coli* isolates from German soldiers after returning predominantly from deployment sites in the African Sahel region, as well as from other deployment settings, were subjected to whole-genome sequencing (WGS). The isolates were collected between 2007 and 2016; a majority of 39 out 51 (76.5%) were phenotypically and superficially genotypically assessed during a previous study [8]. At the end of 2016, the screening and collection of resistant bacteria from deployment returnees halted because the colonization risk was found to be similar to the situation in Germany [8], and different assessments indicated declining colonization rates after returning to low-risk settings [7,71,72]. Relatively high colonization rates among returnees from the Sudan and South Sudan regions [8] most likely resulted from lacking access to field camp infrastructure, because the deployed soldiers operated as UN (United Nations) observers who bought their food and beverages on the local market. A previous assessment [73] demonstrated the particular vulnerability of UN observers in Sudan to fecal–orally transmitted microorganisms, and they frequently suffered from travelers’ diarrhea [74], which is an independent risk factor for enteric colonization with resistant Enterobacterales [75].

In the study presented here, all third-generation cephalosporine-resistant *E. coli* strains from military returnees were assessed by cgMLST (core genome multi-locus sequence typing) and traditional MLST for clonal relationship. Secondly, all isolates were analyzed for the abundance of highly virulent *E. coli* clonal lineages [76], for genotypic and phenotypic antimicrobial resistance and molecular virulence determinants. By doing so, the assessment served as a small sentinel for settings where the access to molecular epidemiological tools and resources is scarce, with a particular focus on the Sahel region.

## 2. Materials and Methods

### 2.1. Strain Collection

The 51 assessed third-generation cephalosporine-resistant *E. coli* strains were isolated from stool samples of 51 out of 961 soldiers during returnee screenings 2–3 months after deployment at predominantly tropical deployment sites within the study interval from 2007 to 2016. The deployment sites comprised Afghanistan (*n* = 175), Argentina (*n* = 2), Belize (*n* = 5), Bosnia and Herzegovina (*n* = 1), Brazil (*n* = 2), the Central African Republic (*n* = 1), China (*n* = 1), the Democratic Republic of the Congo (*n* = 112), Djibouti (*n* = 20), Egypt (*n* = 1), Ethiopia (*n* = 3), French Guyana (*n* = 7), Gabon (*n* = 6), Ghana (*n* = 10), India & Nepal (*n* = 1), Indonesia (*n* = 4), Iraq (*n* = 10), Jamaica (*n* = 1), Kenya (*n* = 3), Kosovo (*n* = 44), Lebanon (*n* = 4), Liberia (*n* = 6), Madeira (*n* = 1), Mali (*n* = 89), Malta (*n* = 1), Morocco (*n* = 1), Nigeria (*n* = 21), not-further-defined African destinations (*n* = 3), not-further-specified regions in the Indian Ocean (*n* = 1), Pakistan (*n* = 2), Panama (*n* = 1), Senegal (*n* = 5), Somalia (*n* = 1), South Africa (*n* = 1), South Sudan (*n* = 35), Sudan (*n* = 202), Sudan and Uganda (*n* = 22), Tanzania (*n* = 7), Thailand (*n* = 6), Uganda (*n* = 41), the United States of America/Hawaii (*n* = 1), unknown or multiple deployment settings (*n* = 21), Uzbekistan (*n* = 73), Venezuela (*n* = 1), Vietnam (*n* = 2), Western Sahara (*n* = 3), and Zimbabwe (*n* = 1) [8]. Although not specifically assessed in this study, German soldiers are usually deployed from the home country and not from other deployment sites. There were no exclusion criteria; all returnees who provided stool samples were included in the screening.

In line with the ethical requirements for this study demanding full anonymization, no returnee-specific details such as age, sex, duration of deployment, and specific mission can be provided. Several of the deployed contingents were so small that respective details might have negatively interfered with the need for anonymization.

### 2.2. Mass-Spectrometry Based Species Identification and Phenotypic Resistance Testing

Confirmation of the strain identity on a species level was performed by matrix-assisted laser desorption ionization time-of-flight mass spectrometry (MALDI TOF MS) using a Bruker MALDI Biotyper^TM^ MBT^TM^ (Bruker Daltonik GmbH, Bremen, Germany) smart mass spectrometer and the software MBT compass version 4.1. Antimicrobial susceptibility was measured with the AST-N214 and AST N-248 Vitek-2 cards of a VITEK-II system (version 9.03, BioMérieux, Marcy-l’Étoile, France); the interpretation was based on the European Committee on Antimicrobial Susceptibility Testing (EUCAST) standards version 11.0.

### 2.3. Whole-Genome Sequencing and Bioinformatics

Total DNA of all strains grown overnight in enriched thioglycolate broth was extracted using the Pathogen Universal Protocol on a MagNA Pure 96 instrument (Roche, Mannheim, Germany). In brief, 1 mL of bacterial culture was centrifuged, and the resulting pellet was resuspended in 100 µL of phosphate-buffered saline. The samples were mixed with 100 µL of MagNA Pure Bacterial Lysis Buffer (Roche, Basel, Switzerland) and 20 µL of Proteinase K (Roche, Basel, Switzerland), and then incubated at 65 °C for 10 min. The lysates were then used for purification of total DNA. The concentration and purity of the extracted gDNA was analyzed using a Nanodrop ONE instrument (Thermo Fisher Scientific, Wilmington, DE, USA). The gDNA was sequenced on an Illumina MiSeq sequencer (Illumina, San Diego, CA, USA) at the Institute for Infection Control and Infectious Diseases at the University Medical Center Göttingen (UMG) generating 2 × 250 bp paired reads and aiming at 100% coverage using V3 chemistry. The NEBnext^®^ Ultra^TM^ II FS DNA Library Prep Kit for Illumina (New England Biolabs, NEB, Ipswich, Massachusetts, USA) was used for library preparation. All raw data who passed the quality control using FASTQC v.0.11.4 [77,78] were included in the bioinformatic analysis. KRAKEN2 v.2.0.8-beta [79] was applied for taxonomic classification and contamination check of raw reads. Raw reads were then trimmed using trimmomatic v0.36 [80] for subsequent phylogenetic analysis. Core genome multi-locus sequence typing (cgMLST) analysis was conducted applying the commercial software SeqSphere+ v. 8.4.1. (Ridom GmbH, Münster, Germany) [81] with NC_000913.3 (*E. coli*) as the reference genome. Assessment of read data and adapter control using FASTQC and subsequent genome assembly using the internally provided assembler Velvet (default settings) were elements of the software pipeline. The *E. coli* cgMLST typing scheme including 2515 core genes provided by the software was used to generate a minimum spanning tree (MST). To become included in the phylogenetic assessment, each sample required a proportion of “good cgMLST targets” higher than 90%. If appropriate, the SeqSphere software automatically assigned novel cgMLST-based complex types (CT). Additionally, the software provided the sequence type (ST) according to the *E. coli* Warwick typing scheme [82]. In the case of allele differences less than 10, clonal identity of the isolates was accepted and defined as a cluster. Next to the abovementioned procedure, raw sequence data were assembled with SPAdes v3.13.11 [83], enabling the careful option. With an in-house script, scaffolds shorter than 500 bp or with a coverage smaller than 10 were sorted out. A screening for virulence factor genes in SPAdes assembly files was conducted using Abricate v.0.9.9 [84] and VFDB (updated last 19 October 2022) [85] as reference databases. Accordingly, virulence factor assessment was focused on pathotype-associated toxins and effectors as described recently [86], i.e., heat-labile enterotoxin (LT), Shiga toxin (Stx), cytolethal distending toxin (CDT), *Shigella* enterotoxin 1 (ShET1), urease, EspC, EspP, hemoglobin-binding protease (Tsh), Pet, Pic, Sat, SepA, SigA, cycle-inhibiting factor (Cif), EspF, EspH, Map, Tir, IpaA, IpaB, IpaC, IpaH, IpgD, VirA, StcE, HylA, Ehx, cytotoxic necrotizing factors (CNF-1, -2), LifA/Efa, Shigella enterotoxin 2 (ShET2), heat-stable enterotoxin a (STa), heat-stable enterotoxin b (STb), and EAST. Lastly, raw data were uploaded to ResFinder 4.1 (https://cge.food.dtu.dk/services/ResFinder/ (accessed on 10 December 2022)) [87], using *Escherichia coli* as selected species and screening for acquired antimicrobial resistance genes to define WGS-predicted phenotypes of resistance against different antimicrobials using default settings (%ID > 90, minimum length > 60%).

### 2.4. Ethics

Ethical clearance for the assessment of the surveillance data in an anonymized way without requirement of informed consent was provided by the ethics committee of the medical association of Rheinland-Pfalz (reference number 2021-16003, provided on 30 July 2021), the ethics committee of the University Medicine Rostock (registration number A2015-0077, 22 June 2015) in line with national and ICH-GCP guidelines, and the ethics committee of the medical association of Hamburg (reference number 2021-300097-WF, provided on 1 November 2021) in line with national German laws. The study was conducted according to the guidelines of the Declaration of Helsinki.

## 3. Results

### 3.1. Details on the Origin of the Assessed E. coli Strains

The identified 51 out of 961 (5.3%) returnees with *E. coli* with resistance against third-generation cephalosporines returned from predominantly subtropical or tropical deployments to Afghanistan (3/175, 1.7%), the Democratic Republic of Congo (3/112, 2.7%), Djibouti (1/20, 5.0%), Ethiopia (1/3, 33.3%), Ghana (1/10, 10.0%), India and Nepal, (1/1, 100.0%), Iraq (2/10, 20.0%), Lebanon (1/4, 25.0%), Mali (8/89, 9.0%), Nigeria (2/21, 9.5%), not-further-defined African destinations (1/3, 33.3%), South Sudan (7/35, 20%), Sudan (9/202, 4.5%), Sudan and Uganda (2/22, 9.1%), Tanzania (1/7, 14.3%), Thailand (1/6, 16.7%), Uganda (3/41, 7.3%), unknown or multiple deployment settings (1/21, 4.8%), and Uzbekistan (3/73, 4.1%). Over the years of the assessment from 2007 to 2016, the average colonization rate of the returnees varied between 0% and 18.7% [8]. None of the isolates included in this assessment was associated with a clinically apparent infection. Returnees from the Sahel region (Djibouti, Mali, South Sudan, Sudan, Sudan, and Uganda) comprised 27/51 (52.9%) and, thus, more than the half of the individuals with proven colonization with cephalosporine-resistant *E. coli*.

### 3.2. Phenotypic Resistance Patterns of the E. coli Isolates with Resistance against Third-Generation Cephalosporines against Carbapenems, Gentamicin, Ciprofloxacin, Moxifloxacin, and Trimethoprim–Sulfamethoxazole

All assessed ESBL-producing *E. coli* strains were susceptible to carbapenems. Focusing on non-beta-lactam-antibiotics, 35/51 (68.6%) strains were susceptible to and 16/51 (31.4%) strains were resistant against gentamicin. For ciprofloxacin, 22/51 (43.1%) strains were susceptible, 3/51 (5.9%) strains were susceptible at increased dose, and 26/51 (51.0%) strains were resistant; for moxifloxacin, 13/51 (25.5%) strains were susceptible and 38/51 (74.5%) strains were resistant. Regarding trimethoprim–sulfamethoxazole, 15/51 (29.4%) strains were susceptible and 36/51 (70.6%) strains were resistant. Susceptibility results stratified by the abundance of beta-lactamases are provided in Table 1. In addition, minimum inhibitory concentration (MIC) details for each isolate are displayed in the Appendix A (Table A1).

### 3.3. Identified Clonal Lineages, Resistance Determinants, as Well as Virulence Determinants, and Their Distribution on the Deployment Sites in the Sahel Regions and in Other Deployment Settings

As shown in Table 2, a high variety of 29 different sequence types (STs) according to the Warwick University scheme were identified. Focusing on STs which occurred more than once, ST10 was isolated from returnees from Afghanistan (*n* = 1) and Sudan (*n* = 1), ST38 was isolated from returnees from Afghanistan (*n* = 1), Mali (*n* = 3), Nigeria (*n* = 1), South Sudan (*n* = 3), and Sudan (*n* = 1), ST48 was isolated from returnees from Iraq (*n* = 1) and from unknown or multiple deployment sites (*n* = 1), ST131 was isolated from returnees from Iraq (*n* = 1), Mali (*n* = 1), Sudan (*n* = 3), and Tanzania (*n* = 1), ST167 was isolated from returnees from Mali (*n* = 1) and Uzbekistan (*n* = 1), ST405 was isolated from returnees from Ethiopia (*n* = 1) and Sudan (*n* = 1), ST617 was isolated from returnees from the Democratic Republic of the Congo (*n* = 1), Uganda (*n* = 1), and Uzbekistan (*n* = 1), ST641 was isolated from returnees from the Democratic Republic of the Congo (*n* = 1) and Lebanon (*n* = 1), ST648 was isolated from returnees from Mali (*n* = 1) and South Sudan (*n* = 1), and ST1312 was isolated from returnees from Afghanistan (*n* = 1) and India and Nepal (*n* = 1).

Focusing on the ST types from returnees from the Sahel region, ST5942 (*n* = 1) was isolated from a returnee from Djibouti, ST38 (*n* = 3), ST101 (*n* = 1), ST131 (*n* = 1), ST167 (*n* = 1), ST450 (*n* = 1), and ST648 (*n* = 1) were isolated from returnees from Mali, ST38 (*n* = 3), ST616 (*n* = 1), ST636 (*n* = 1), ST648 (*n* = 1), and ST2624 (*n* = 1) were isolated from returnees from South Sudan, ST10 (*n* = 1), ST38 (*n* = 1), ST131 (*n* = 3), ST405 (*n* = 1), ST410 (*n* = 1), ST767 (*n* = 1), and ST940 (*n* = 1) were isolated from returnees from Sudan, and ST394 (*n* = 1) and ST4305 (*n* = 1) were isolated from returnees from Sudan and Uganda.

Focusing on clonal lineages which have been previously associated with virulence [76] but without consideration of virulence factor detection, ST88 was isolated from a returnee from Nigeria. Details on the detection of ST10 and ST131 were provided above.

**Figure 1:**
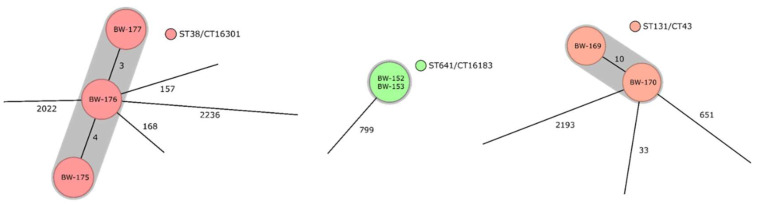
Observed clusters of closely related isolates. Numbers between the nodes indicate allele differences of the cgMLST analysis between the different samples.

Close genetic relatedness below the sequence type level with a maximum of 10 discrepant core gene alleles was observed for the strains BW-152 and BW-153 of the sequence type ST641, for the strains BW-169 and BW-170 of the sequence type ST131, and for the strains BW-175, BW-176, and BW-177 of the sequence type ST38 (Figure 1). While no association could be shown for the soldiers carrying BW-152 and BW-153, the soldiers carrying BW-152 and BW-153 were both deployed to Darfur, but with a time difference of 2 years. The soldiers carrying BW175, BW176, and BW177 were deployed in parallel in Darfur and Juba; thus, personal contacts potentially resulting in transmission events or exposure to the same source during deployment is at least hypothetically possible, although definite proof is not available. In particular, there were no records unambiguously proving personal contact.

As shown in Table 2, the most frequently detected ESBL-phenotype associated beta-lactamase was *bla*_CTX-M-15_ (*n* = 35), followed by *bla*_CTX-M-1_ (*n* = 6), *bla*_CTX-M-27_ (*n* = 5), *bla*_CTX-M-14_ (*n* = 2), *bla*_TEM-169_ (*n* = 2), *bla*_CTX-M-99_-like (*n* = 1), *bla*_CTX-M-125_ (*n* = 1), *bla*_SHV-12_ (*n* = 1), and *bla*_SHV-13_ (*n* = 1)_,_ along with other, not ESBL-associated beta-lactamases. When focusing on the Sahel region, a *bla*_SHV-12_-carrying isolate was imported from Djibouti, isolates carrying *bla*_CTX-M-1_ (*n* = 1), *bla*_CTX-M-15_ (*n* = 5), and *bla*_CTX-M-27_ (*n* = 2) were imported from Mali, isolates carrying *bla*_CTX-M-14_ (*n* = 1), *bla*_CTX-M-15_ (*n* = 2), *bla*_CTX-M-15_ and *bla*_TEM-169_ (*n* = 2), *bla*_CTX-M-99_-like (*n* = 1), and *bla*_CTX-M-125_ (*n* = 1) were imported from South Sudan, isolates carrying *bla*_CTX-M-1_ (*n* = 1), *bla*_CTX-M-15_ (*n* = 6), and *bla*_CTX-M-27_ (*n* = 2) were imported from Sudan, and isolates carrying *bla*_CTX-M-15_ (*n* = 2) were imported from Sudan and Uganda. Other frequently detected acquired antimicrobial resistance (AMR) genes encoded various enzymes mediating resistance against aminoglycosides, cotrimoxazole (*dfrA*- and *sul*-genes), and tetracycline (*tet*-genes). Additional AMR genes were less frequently observed. Details are provided in Table 2.

In Table 3, recorded minimum inhibitory concentrations for gentamicin, trimethoprim/sulfamethoxazole, ciprofloxacin, and moxifloxacin measured with isolates with and without detected specific resistance genes are compared. As shown in detail, detection of specific resistance genes was associated with higher minimum inhibitory concentration values.

As shown in Table 4, the screened virulence-associated genes were recorded in 16 out of 51 (31.4%) isolates including multiple detections per isolate. The *cnf-1* gene encoding the cytotoxic necrotizing factor was observed in an isolate from a returnee from the Democratic Republic of the Congo, the *east1* gene encoding a heat-stable enterotoxin was observed in isolates from returnees from Afghanistan (*n* = 1), the Democratic Republic of the Congo (*n* = 1), Iraq (*n* = 1), Mali (*n* = 2), not-further-defined African destinations (*n* = 1), Sudan (*n* = 2), Sudan and Uganda (*n* = 2), and Uzbekistan (*n* = 1), the *hlyA* gene encoding the hemolysin A was observed in an isolate from a returnee from Thailand, the *sat* gene encoding the secreted autotransporter toxin was observed in isolates from returnees from Iraq (*n* = 1), Mali (*n* = 2), South Sudan (*n* = 1), and Sudan (*n* = 1), and the *tsh* gene encoding the hemoglobin-binding protease was observed in an isolate from a returnee from Sudan. Please see Table 4 for details regarding the specific distribution of the virulence genes on the isolates. Focusing on the Sahel region, identified virulence genes comprised *east1*, *sat*, and *tsh* in declining order of frequency of occurrence.

## 4. Discussion

The study was conducted to provide a next-generation sequencing-based characterization of third-generation cephalosporine-resistant *E. coli* isolated from stool samples of returnees from military deployments with particular focus on the African Sahel region in the course of the assessment interval. In doing so, the assessment was meant to broaden previously collected information [8] by providing an in-depth analysis of clonal distribution, as well as of molecular resistance and virulence determinants.

Not surprisingly, the majority of resistant *E. coli* were imported from the African Sahel region. On the regional deployments, high exposure had to be considered as likely due to local partnering during training missions such as in Mali [51,52,53] or due to living under local conditions as it was the case for the United Nations observers in South Sudan and Sudan [73]. The sequence types ST38, ST131, and ST648, which were repeatedly isolated from returnees from the Sahel region, are well known from other epidemiological assessments from various sub-Saharan regions [88,89,90,91,92,93,94,95,96,97,98,99].

As expected, a broad variety of different sequence types was observed among the isolates. Of note, some of the recorded sequence types were previously associated with *E. coli* showing increased pathogenicity [76], such as ST10 with enteropathogenic *E. coli*, ST88 with extra-intestinal pathogenic *E. coli*, avian pathogenic *E. coli*, and enteropathogenic *E. coli*, and ST131 with adherent invasive *E. coli*, uropathogenic *E. coli*, and extra-intestinal pathogenic *E. coli*. Of note, however, no suggestive virulence factors could be detected for the ST88 isolate. In soldiers deployed to the Sahel region, the abundance of ST10 and ST131 was confirmed.

Surprisingly, a close phylogenetic relationship could be confirmed for a small number of isolates from three clusters, suggesting nosocomial transmission or common sources of transmission. In line with the retrospective character of the assessment, likely modes of transmission could only partly be reconstructed. As expected [51,52,53,73], the deployments in the Sahel region were again particularly affected.

ESBL-type resistance was mediated by a variety of resistance genes with an overwhelming predominance of CTX-M-type enzymes followed by TEM-type and SHV-type mechanisms. The details provided in this study may contribute to previous epidemiological information from the Sahel region [42,43,44,45,46,47,48,49,50,51,52,53,54,55,56,57,58,59,60,61,62,63,64,65,66,67,68,69,70]. In line with the phenotypically observed high resistance rates, high numbers of acquired resistance genes mediating aminoglycoside and cotrimoxazole resistance were also recorded.

As expected due to the sampling between 2 and 3 months after deployment from healthy individuals [8], diarrheagenic *E. coli* were not observed among the isolates. Altogether, virulence factor genes which are typically associated with pathogenic *E. coli* [86] such as meningitis-associated *E. coli*, uropathogenic *E. coli*, necrotoxic *E. coli*, extra-intestinal pathogenic *E. coli*, and avian pathogenic *E. coli* were observed in only a minority of isolates.

The study had a number of limitations. First, the assessment provided only a historic overview, because the screening was ceased after 2016 for organizational reasons. Second, next-generation sequencing had to be restricted to short-read assessments due to funding restrictions. Accordingly, reconstruction of resistance gene-carrying plasmids was not attempted. Third, no conclusions can be drawn from the results on the prevalence of ESBL-positive *E. coli* directly after deployment. As repeatedly shown [71,72], a tremendous decline in detectable colonization with ESBL-positive Enterobacterales can be expected within the first months after return. Fourth, no detailed patient-specific data could be shown for ethical reasons, because such details might have negatively interfered with the need for anonymization in the case of small deployments. Fifth, although deployment-association of the acquisition of the ESBL-positive Enterobacterales is hypothetically possible, it is not definitely proven in this study. Soldiers can, in principle, act as vehicles in dissiminating resistant bacteria in the case of transfer from one deployment region to the other or back to their home country. A very prominent historical incident of such a pathogen transfer was the cholera epidemic which affected Haiti from October 2010 to February 2019 as a consequence of the importation of *Vibrio cholerae* by UN peacekeepers arriving form Nepal [100]. However, the study design chosen for this assessment is unsuitable for verifying or falsifying such importation events.

## 5. Conclusions

In summary, the study provided a first next-generation sequencing-based assessment of third-generation cephalosporine resistance in *E. coli* isolates imported by German soldiers from abroad deployments. Respective isolations were predominantly observed from soldiers after deployment in the African Sahel regions. A high variety of sequence types and ESBL-resistance mediating genes was recorded, while virulence genes were only scarcely detected.

## Figures and Tables

**Table 1:** Antimicrobial susceptibility of all isolates stratified by beta-lactamase detection and deployment site.

Beta- Lactamase (BL)	No.	Deployment Sites	MIC Value	MIC (mg/L)											
				CTX	CTZ	CPD	FEP	ERT	MER	TZP	CTM	CIP	GEN	FOF	AZT
*bla*_CTX-M-15_, *bla*_TEM-1B_	*n* = 12	Afghanistan, Congo, India/Nepal, Iraq, Mali, Sudan, Uganda	Median	≥64	4	≥8	2	≤0.5	≤0.25	≤4	≥320	≤0.25	≤1	≤16	16
			Range	≥64 to ≥64	≤1 to 16	≥8 to ≥8	≤1 to ≥64	≤0.5 to ≤0.5	≤0.25 to ≤0.25	≤4 to 16	≤20 to >320	≤0.25 to ≥4	≤1 to ≥16	≤16 to 64	≤1 to >64
*bla*_CTX-M-15_, *bla*_TEM-1B, _ *bla*_OXA-1_	*n* = 9	Congo, Mali, Nigeria, Sudan, Uganda, Uzbekistan	Median	≥64	≥64	≥8	8	≤0.5	≤0.25	8	≥320	≥ 4	≤1	≤16	≥64
			Range	≥64 to ≥64	4 to ≥64	≥8 to ≥8	2 to ≥64	≤0.5 to ≤0.5	≤0.25 to ≤0.25	≤4 to >128	≤20 to >320	≤0.25 to ≥4	≤1 to ≥16	≤16 to 64	16 to ≥64
*bla*_CTX-M-15_, *bla*_OXA-1_	*n* = 7	Ethiopia, Tanzania, Sudan, Uzbekistan	Median	≥64	16	≥8	4	≤0.5	≤0.25	8	≥320	≥ 4	≤1	≤16	32
			Range	4 to ≥64	≤1 to ≥64	≥8 to ≥8	≤1 to ≥64	≤0.5 to ≤0.5	≤0.25 to ≤0.25	8 to ≥64	≤20 to >320	1 to ≥4	≤1 to ≥16	≤16 to 64	≤1 to ≥64
*bla*_CTX-M-15_ with or without other BL	*n* = 7	Mali, Sudan, Uganda	Median	≥64	16	≥8	4	≤0.5	≤0.25	≤4	≥320	0.5	≤1	≤16	16
			Range	≥64 to ≥64	≤1 to 16	≥8 to ≥8	≤1 to ≥64	≤0.5 to ≤0.5	≤0.25 to ≤0.25	≤4 to 64	≤20 to >320	≤0.25 to ≥4	≤1 to ≤1	≤16 to 64	2 to 16
*bla*_CTX-M-1_ with or without other BL	*n* = 6	Congo, Ghana, Lebanon, Mali, Nigeria, Sudan	Median	≥64	4	≥8	2	≤0.5	≤0.25	≤4	≤20	≤0.25	≤1	≤16	16
			Range	≥64 to ≥64	≤1 to 4	≥8 to ≥8	2 to 4	≤0.5 to ≤0.5	≤0.25 to ≤0.25	≤4 to ≤4	≤20 to >320	≤0.25 to ≥4	≤1 to ≥16	≤16 to 64	2 to ≥64
*bla*_CTX-M-27_	*n* = 5	Mali, Sudan, Thailand	Median	≥64	4	≥8	2	≤0.5	≤0.25	≤4	≥320	≤0.25	≤1	≤16	4
			Range	≥64 to ≥64	4 to 4	≥8 to ≥8	≤1 to 2	≤0.5 to ≤0.5	≤0.25 to ≤0.25	≤4 to ≤4	≤20 to >320	≤0.25 to ≥4	≤1 to ≤1	≤16 to ≤16	2 to 16
Other BL	*n* = 5	Afghanistan, Djibouti, Sudan	Median	≥64	≤1	≥8	≤1	≤0.5	≤0.25	≤4	≥320	1	≥ 16	≤16	16
			Range	≥64 to ≥64	≤1 to ≥64	4 to ≥8	≤1 to 2	≤0.5 to ≤0.5	≤0.25 to ≤0.25	≤4 to 16	≤20 to >320	≤0.25 to ≥4	≤1 to ≥16	≤16 to 64	2 to 16

Abbreviations: AZT = aztreonam, CIP = ciprofloxacin, CPD = cefpodoxime, CTM = trimethoprim/sulfamethoxazole, CTX = cefotaxime, CTZ = ceftazidime, ERT = ertapenem, FEP = cefepime, FOF = fosfomycin, GEN = gentamicin, MER = meropenem, TZP = piperacillin/tazobactam.

**Table 2:** Sequence types (STs) according to the Warwick University scheme and acquired molecular resistance mechanisms in the assessed ESBL-positive *E. coli* isolates detected by Resfinder with a sequence identity and coverage of ≥99%.

Anonymized Strain Identity	Year of Isolation	Deployment Site	Sequence Type (ST) According to the Warwick University Scheme	Beta-Lactamase Genes (ESBL-Associated Underlined)	Genes Mediating Aminoglycoside Resistance	Genes Mediating Chloramphenicol Resistance	Genes Mediating Resistance against Trimethoprim or Sulfonamides	Genes Mediating Resistance against Macrolides or Lincosamides or Streptogramins	Genes Mediating Resistance against Fluoroquinolones	Genes Mediating Resistance against Tetracyclines	Genes Mediating Tolerance against Disinfectants
BW-151	2007	Uzbekistan	617	*bla*_CTX-M-15_, *bla*_OXA-1_	*aac(6*2019*)-Ib-cr*, *aadA5*, *aph(3*″*)-Ib*, *aph(6)-Id*	-	*dfrA17*, *sul1*, *sul2*	-	*aac(6*′*)-Ib-cr*	*tet(B)*	*sitABCD*
BW-152	2007	Democratic Republic of the Congo	641	*bla*_CTX-M-1_, *bla*_TEM-1B_	*aadA1*	*cmlA1*	*sul3*	*mph(A)*	-	*tet(B)*	-
BW-153	2007	Lebanon	641	*bla*_CTX-M-1_, *bla*_TEM-1B_	*aadA1*	*cmlA1*	*sul3*	*mph(A)*	-	*tet(B)*	-
BW-154	2007	Uzbekistan	167	*bla*_CTX-M-15_, *bla*_OXA-1_	*aac(3)-IIa*, *aac(6*′*)-Ib-cr*, *aadA5*, *aph(3*″*)-Ib*, *aph(6)-Id*	-	*dfrA17*, *sul2*	*erm(B)*, *mph(A)*	*aac(6*′*)-Ib-cr*	*tet(A)*	*sitABCD*
BW-155	2007	Afghanistan	1312	*bla*_CTX-M-15_, *bla*_TEM-1B_	*aac(3)-IId*, *aadA2*, *aph(3*″*)-Ib*, *aph(6)-Id*	*catA1*	*dfrA12*, *sul1*, *sul2*	*mph(A)*	-	*tet(B)*	*sitABCD*
BW-156	2007	Democratic Republic of the Congo	70	*bla*_CTX-M-15_, *bla*_TEM-1B_	*aph(3*″*)-Ib, aph(6)-Id*	*catA1*	*dfrA7*, *sul1*, *sul2*	-	-	*tet(B)*	-
BW-157	2007	Uzbekistan	43	*bla*_CTX-M-15_, *bla*_OXA-1_, *bla*_TEM-1B_	*aac(6*′*)-Ib-cr*, *aadA1*, *aph(3*″*)-Ib*, *aph(6)-Id*	-	*dfrA1*, *sul2*	-	*aac(6*′*)-Ib-cr*	*tet(A)*, *tet(B)*	-
BW-158	2007	Sudan	131	*bla*_CTX-M-15_, *bla*_OXA-1_	*aac(6*′*)-Ib-cr*, *aadA5*	-	*dfrA17*, *sul1*	*mph(A)*	*aac(6*′*)-Ib-cr*	-	-
BW-159	2008	Sudan	38	*bla*_CTX-M-15_, *bla*_TEM-1B_	*aadA1*, *aph(6)-Id*	-	*dfrA1*, *sul2*	-	-	-	-
BW-160	2009	Sudan	410	*bla*_CTX-M-15_, *bla*_OXA-1_, *bla*_TEM-1B_	*aac(6*′*)-Ib-cr*, *aph(3*″*)-Ib*, *aph(6)-Id*	-	*dfrA14*, *sul2*	*mph(A)*	*aac(6*′*)-Ib-cr*	*tet(A)*	*sitABCD*
BW-161	2009	Ghana	446	*bla* _CTX-M-1_	-	-	-	-	-	-	-
BW-162	2009	Sudan	405	*bla*_CTX-M-15_, *bla*_OXA-1_	*aac(6*′*)-Ib-cr*	-	-	-	*aac(6*′*)-Ib-cr*	*tet(B)*	-
BW-163	2010	Sudan	10	*bla*_CTX-M-15_, *bla*_OXA-1_	*aac(6*′*)-Ib-cr*	-	-	-	*aac(6*′*)-Ib-cr*	*tet(B)*	-
BW-164	2010	Afghanistan	38	*bla* _CTX-M-14_	*aadA2*, *aph(3*″*)-Ib*, aph(6)-Id	-	*dfrA12*, *sul2*	*mph(A)*	-	-	-
BW-165	2010	Sudan	767	*bla* _CTX-M-1_	*aph(3*″*)-Ib*, *aph(6)-Id*	-	*sul2*	*mph(A)*	-	-	*sitABCD*
BW-166	2010	Thailand	3037	*bla* _CTX-M-27_	-	-	-	-	-	*tet(A)*	-
BW-167	2011	Afghanistan	10	*bla*_CTX-M-15_, *bla*_TEM-1B_	*aadA1*, *aph(3*″*)-Ib*, *aph(6)-Id*	*catA1*	*dfrA1*, *sul2*	-	-	*tet(A)*	-
BW-168	2011	Sudan	940	*bla* _CTX-M-15_	*aph(3*″*)-Ib*, *aph(6)-Id*	-	*dfrA1*, *sul2*	-	-	*tet(B)*	-
BW-169	2011	Sudan	131	*bla* _CTX-M-27_	*aph(3*″*)-Ib*, *aph(6)-Id*	-	*sul1*, *sul2*	*mph(A)*	-	-	-
BW-170	2013	Sudan	131	*bla* _CTX-M-27_	*aadA5*, *aph(3*″*)-Ib*, *aph(6)-Id*	-	*dfrA17*, *sul1*, *sul2*	*mph(A)*	-	-	-
BW-171	2013	Djibouti	5942	*bla*_SHV-12_, *bla*_TEM-1B_	*aac(3)-IV*, *aadA2*, *aph(3*″*)-Ib*, *aph(4)-Ia*, *aph(6)-Id*	*-*	-	-	*qnrS1*	*tet(B)*	--
BW-172	2013	Tanzania	131	*bla*_CTX-M-15_, *bla*_OXA-1_	*aac(6*′*)-Ib-cr*, *aadA5*, *aph(3*″*)-Ib*, *aph(6)-Id*	-	*dfrA17*, *sul2*	*mph(A)*	*aac(6*′*)-Ib-cr*	-	-
BW-173	2014	Uganda	656	*bla*_CTX-M-15_, *bla*_SHV-13_, *bla*_TEM-1B_	*aph(3*″*)-Ib*, *aph(6)-Id*	-	*dfrA14*, *sul2*	*mph(A)*	-	*tet(B)*	*sitABCD*
BW-174	2014	Uganda	448	*bla*_CTX-M-15_, *bla*_OXA-1_, *bla*_TEM-1B_	*aac(3)-IIa*, *aac(6*′*)-Ib-cr*, *aadA5*, *aph(3*″*)-Ib*, *aph(3*′*)-Ia*, *aph(6)-Id*	-	*dfrA17*, *sul2*	*mph(A)*	*aac(6*′*)-Ib-cr*	*tet(A)*, *tet(B)*	-
BW-175	2014	South Sudan	38	*bla*_CTX-M-125_, *bla*_DHA-1_, *bla*_TEM-1B_	*aac(3)-IIa*, *aadA5*	-	*dfrA17*	*mph(A)*	*qnrB4*	-	-
BW-176	2014	South Sudan	38	*bla*_CTX-M-99_-like (<99% sequence homology), *bla*_TEM-1B_	*aac(3)-IIa*, *aadA5*	-	*dfrA17*	*mph(A)*	-	-	-
BW-177	2014	South Sudan	38	*bla*_CTX-M-14_, *bla*_TEM-1B_	*aac(3)-IId*, *aadA5*	-	*dfrA17*, *sul1*	*mph(A)*	-	-	-
BW-178	2014	South Sudan	636	*bla*_CTX-M-15_, *bla*_TEM-169_, *bla*_TEM-1B_	*aadA1*, *aph(3*″*)-Ib*, *aph(6)-Id*	-	*dfrA1*, *sul2*	*mph(A)*	-	*tet(B)*	-
BW-179	2014	Uganda	617	*bla*_CTX-M-15_, *bla*_OXA-1_, *bla*_TEM-1B_	*aac(3)-IIa*, *aac(6*′*)-Ib-cr*, *aadA5*	*catA1*	*dfrA17*	*mph(A)*	*aac(6*′*)-Ib-cr*	*tet(B)*	*sitABCD*
BW-180	2013	South Sudan	2624	*bla*_CTX-M-15_, *bla*_TEM-1B_	*aph(3*″*)-Ib*, *aph(6)-Id*	-	*sul2*	-	*qnrS1*	-	-
BW-181	2014	South Sudan	616	*bla*_CTX-M-15_, *bla*_TEM-169_	*aph(3*″*)-Ib*, *aph(6)-Id*	-	*dfrA14*, *sul2*	-	*qnrS1*	*tet(A)*	-
BW-182	2014	Nigeria	38	*bla*_CTX-M-15_, *bla*_OXA-1_, *bla*_TEM-1B_	*aac(6*′*)-Ib-cr*, *aadA1*, *aph(3*″*)-Ib*, *aph(6)-Id*	*catA1*	*dfrA1*, *sul2*	-	*aac(6*′*)-Ib-cr*	*tet(A)*, *tet(D)*	-
BW-183	2014	Democratic Republic of the Congo	617	*bla*_CTX-M-15_, *bla*_OXA-1_, *bla*_TEM-1B_	*aac(3)-IIa*, *aac(6*′*)-Ib-cr*, *aadA5*, *aph(3*″*)-Ib*, *aph(6)-Id*	*catA1*	*dfrA17*, *sul1*, *sul2*	*mph(A)*	*aac(6*′*)-Ib-cr*	*tet(B)*	*sitABCD*
BW-184	2014	Mali	38	*bla* _CTX-M-27_	*aadA5*, *aph(3*″*)-Ib*, *aph(6)-Id*	-	*dfrA17*, *sul1*, *sul2*	*mph(A*)	-	-	-
BW-185	2014	Mali	101	*bla* _CTX-M-1_	*aadA5*	-	*dfrA17*, *sul2*	-	-	-	*sitABCD*
BW-186	2014	South Sudan	648	*bla*_CTX-M-15_, *bla*_OXA-1_, *bla*_TEM-1B_	*aac(3)-IIa*, *aac(6*′*)-Ib-cr*, *aadA5*, *aph(3*″*)-Ib*, *aph(6)-Id*	*catA1*	*dfrA17*, *sul2*	*mph(A)*	*aac(6*′*)-Ib-cr*	*tet(B)*	*sitABCD*
BW-187	2015	Mali	38	*bla* _CTX-M-27_	*aadA5*, *aph(3*″*)-Ib*, *aph(6)-Id*	-	*dfrA17*, *sul1*, *sul2*	*mph(A)*	-	-	-
BW-188	2015	not further defined African destinations	677	*bla*_CTX-M-15_, *bla*_TEM-1B_	*aadA1*, *aph(3*″*)-Ib*, *aph(6)-Id*	-	*dfrA1*, *sul1*, *sul2*	-	*qnrS1*	-	-
BW-189	2014	unknown or multiple deployment settings	48	*bla*_CTX-M-15_, *bla*_OXA-1_, *bla*_TEM-1B_	*aac(3)-IIa*, *aac(6*′*)-Ib-cr*, *aadA12*, *aph(3*″*)-Ib*, *aph(6)-Id*	-	*dfrA14*, *dfrA5*, *sul2*	*erm(B)*	*qnrB1*, *aac(6*′*)-Ib-cr*	*tet(A)*	-
BW-192	2016	Sudan & Uganda	4305	*bla*_CTX-M-15_, *bla*_TEM-1B_	*aph(6)-Id*	*dfrA14*, *sul2*	-	*mph(A)*	-	*tet(B)*	-
BW-193	2016	Nigeria	88	*bla* _CTX-M-1_, *bla*_OXA-1_	*aac(3)-IId*, *aadA5*, *ant(2*″*)-Ia*, *aph(*3″)*-Ib*, *aph(3*′*)-Ia*, *aph(6)-Id*	-	*dfrA17*, *dfrA36*, *sul1*	*mph(A)*	-	*tet(B)*	-
BW-194	2016	Mali	167	*bla* _CTX-M-15_	-	*catA1*	-	*erm(B)*, *mph(A)*	-	*tet(B)*	-
BW-195	2016	Mali	131	*bla*_CTX-M-15_, *bla*_TEM-1B_	-	-	-	-	-	-	-
BW-198	2016	Ethiopia	405	*bla*_CTX-M-15_, *bla*_OXA-1_	*aac(3)-IIa*, *aac(6*′*)-Ib-cr*, *aadA5*	*catA1*	*dfrA17*, *sul1*	*mph(A)*	*aac(6*′*)-Ib-cr*	*tet(B)*	
BW-199	2016	Mali	450	*bla*_CTX-M-15_, *bla*_TEM-1B_	*aph(3*″*)-Ib*, *aph(6)-Id*	-	*dfrA14*, *sul2*	-	*qnrS1*	-	-
BW-201	2016	India & Nepal	1312	*bla*_CTX-M-15_, *bla*_TEM-1B_	*aac(3)-IId*, *aph(3*″*)-Ib*, *aph(6)-Id*	*catA1*	*dfrA12*, *dfrA8*, *sul1*, *sul2*	*mph(A)*	-	*tet(B)*	*sitABCD*
BW-203	2016	Sudan & Uganda	394	*bla* _CTX-M-15_	*aph(3*″*)-Ib*, *aph(6)-Id*	-	*dfrA1*, *dfrA7*, *sul2*	-	*qnrS1*	-	-
BW-206	2016	Mali	38	*bla* _CTX-M-15_	*aph(3*″*)-Ib*, *aph(6)-Id*	-	*dfrA1*, *sul2*	-	-	-	-
BW-207	2016	Iraq	131	*bla*_CTX-M-15_, *bla*_TEM-1B_	*aac(3)-IId*, *aadA5*,	-	*dfrA17*, *sul1*		-	-	*sitABCD*
BW-208	2016	Mali	648	*bla*_CTX-M-15_, *bla*_OXA-1_, *bla*_TEM-1B_	*aac(6*′*)-Ib-cr*, *aadA5, aph(3*″*)-Ib*, *aph(6)-Id*	*catB3*	*dfrA17*, *sul2*	-	*aac(6*′*)-Ib-cr*	*tet(B)*	-
BW-209	2016	Iraq	48	*bla*_CTX-M-15_, *bla*_TEM-1B_	*aac(3)-IId*, *aph(3*″*)-Ib*, *aph(6)-Id*	*catA1*	*dfrA12*, *sul1*, *sul2*	*mph(A)*	*qnrS1*	*tet(B)*	*sitABCD*

**Table 3:** Comparison of minimum inhibitory concentrations for gentamicin, trimethoprim/sulfamethoxazole, ciprofloxacin, and moxifloxacin measured with isolates with and without detected specific resistance genes. To allow calculation with the measured values, “≤” and “≥” symbols were ignored.

Antimicrobially Active Drug or Drug Combination	Minimum Inhibitory Concentration in Isolates without Detected Specific Resistance Genes, Mean Value (± Standard Deviation)	Minimum Inhibitory Concentration in Isolates with Detected Specific Resistance Genes, Mean Value (± Standard Deviation)	Significance P *
Gentamicin	1 (±0)	6 (±7.0)	n.e. ^#^
Trimethoprim/sulfamethoxazole	80 (±106.1)	264.2 (±118.1)	0.002
Ciprofloxacin	1.0 (±1.5)	2.6 (±1.7)	0.0006
Moxifloxacin	2.0 (±3.1)	5.4 (±3.3)	0.0002

* Significance P was calculated by applying Mann–Whitney testing with the software GraphPad Instat, version 3.06 (GraphPad Software Inc., La Jolla, CA, USA). ^#^ *p*-Value calculation was not estimable (n.e.) for technical reasons, because the standard deviation in one population was zero.

**Table 4:** Distribution of chosen virulence factor genes (sequence identity ≥99%) within the assessed *Escherichia coli* strains. Only isolates with identified virulence factors are shown.

Anonymized Strain Identity	Year of Isolation	Deployment Site	Cytotoxic Necrotizing Factor (*cnf-1*) Gene ^1^	Heat-Stable Enterotoxin (*east1*) Gene ^2^	Hemolysin A (*hlyA*) Gene ^3^	Secreted Autotransporter Toxin (*sat*) Gene ^4^	Hemoglobin-Binding Protease (*tsh*) Gene ^5^
BW-151	2007	Uzbekistan		X			
BW-156	2007	Democratic Republic of the Congo	X	X			
BW-165	2010	Sudan		X			
BW-166	2010	Thailand			X		
BW-167	2011	Afghanistan		X			
BW-168	2011	Sudan		X			
BW-169	2011	Sudan				X	X
BW-181	2014	South Sudan				X	
BW-184	2014	Mali		X			
BW-187	2015	Mali		X			
BW-188	2015	Not further defined African destinations		X			
BW-192	2016	Sudan and Uganda		X			
BW-195	2016	Mali				X	
BW-199	2016	Mali				X	
BW-203	2016	Sudan and Uganda		X			
BW-207	2016	Iraq		X		X	

^1^ Associated with MNEC (meningitis-associated *E. coli*), UPEC (uropathogenic *E. coli*), and NTEC (necrotoxic *E. coli*); ^2^ associated with various *E. coli* pathotypes; ^3^ associated with UPEC; ^4^ associated with UPEC; ^5^ associated with ExPEC (extra-intestinal pathogenic *E. coli*) and APEC (avian pathogenic *E. coli*).

## Data Availability

All relevant data are presented in the manuscript. Raw data can be made available on reasonable request. Whole-genome sequence data are deposited at the SRA under accession number PRJNA899324. Additional GenBank deposition of sequence data was not performed.

## Appendix A

**Table A1:** Measured minimum inhibitory concentrations (MICs, mg/L) of the assessed ESBL-positive *E. coli* isolates. All isolates were resistant to ampicillin (each isolate MIC of ≥32 mg/L) and susceptible to ertapenem (each isolate MIC of ≤0.5 mg/L).

Anonymized Strain I.D.	Year of Isolation	Deployment Site	ASU	AZT	CTX	CPD	CTZ	CIP	FEP	FOF	GEN	IMI	MER	MOX	TZP	CTM
BW-151	2007	Uzbekistan	≥32	≥64	≥64	≥8	16	≥4	2	≤16	≤1	≤0.25	≤0.25	≥8	8	≥320
BW-152	2007	Democratic Republic of the Congo	≥32	≥64	≥64	≥8	4	≤0.25	2	≤16	≤1	≤0.25	≤0.25	≤0.25	≤4	≤20
BW-153	2007	Lebanon	≥32	16	≥64	≥8	4	≤0.25	2	≤16	≤1	≤0.25	≤0.25	≤0.25	≤4	≤20
BW-154	2007	Uzbekistan	≥32	≥64	≥64	≥8	16	≥4	≥64	≤16	≥16	≤0.25	≤0.25	≥8	64	≥320
BW-155	2007	Afghanistan	16	16	≥64	≥8	8	≤0.25	2	≤16	≥16	≤0.25	≤0.25	≤0.25	≤4	≥320
BW-156	2007	Democratic Republic of the Congo	16	16	≥64	≥8	16	≤0.25	2	≤16	≤1	≤0.25	≤0.25	≤0.25	≤4	≥320
BW-157	2007	Uzbekistan	≥32	≤64	≤64	≥8	16	≥4	8	≤16	≤1	≤0.25	≤0.25	≥8	8	≥320
BW-158	2007	Sudan	≥32	≤1	4	≥8	≤1	≥4	≤1	≤16	≤1	≤0.25	≤0.25	≥8	8	≥320
BW-159	2008	Sudan	16	≥64	≥64	≥8	16	≤0.25	≥64	≤16	≤1	≤0.25	≤0.25	≤0.25	≤4	≥320
BW-160	2009	Sudan	≥32	≥64	≥64	≥8	16	≥4	2	≤16	≤1	≤0.25	≤0.25	≥8	≥128	≥320
BW-161	2009	Ghana	16	≥64	≥64	≥8	≤1	≤0.25	2	≤16	≤1	≤0.25	≤0.25	≤0.25	≤4	≤20
BW-162	2009	Sudan	≥32	≥64	≥64	≥8	≥64	≥4	≥64	≤16	≤1	≤0.25	≤0.25	≥8	16	≤20
BW-163	2010	Sudan	≥32	16	≥64	≥8	16	≥4	2	≤16	≤1	≤0.25	≤0.25	≥8	8	≤20
BW-164	2010	Afghanistan	16	2	≥64	≥8	≤1	≥4	2	≤16	≤2	≤0.25	≤0.25	≥8	≤4	≥320
BW-165	2010	Sudan	16	16	≥64	≥8	4	≤.25	2	≤16	4	≤0.25	≤0.25	≤0.25	≤4	≤20
BW-166	2010	Thailand	≥2	2	≥64	≥8	4	≤0.25	≤1	≤16	≤1	≤0.25	≥0.25	≤0.25	≤4	≤20
BW-167	2011	Afghanistan	16	16	≥64	≥8	4	≤0.25	≤1	≤16	≤1	≤0.25	≤0.25	0.5	≤4	≥320
BW-168	2011	Sudan	16	4	≥64	≥8	16	0.5	4	≤16	≤1	≤0.25	≤0.25	0.5	≤4	≥320
BW-169	2011	Sudan	≤2	4	≥64	≥8	4	≥4	2	≤16	≤1	≤0.25	≥0.25	≥8	≤4	≤20
BW-170	2013	Sudan	≤2	2	≥64	≥8	4	≥4	≤1	≤16	≤1	≤0.25	≤0.25	≥8	≤4	≥320
BW-171	2013	Djibouti	≥32	16	≥64	4	≥64	≥4	≤1	≤16	≥16	≤0.25	≤0.25	≥8	≤4	≤20
BW-172	2013	Tanzania	≥32	16	≥64	≥8	16	1	4	≤16	≤1	≤0.25	≤0.25	1	8	≥320
BW-173	2014	Uganda	16	16	≥64	≥8	4	0.5	2	≤16	≤1	≤0.25	≤0.25	2	≤4	≥320
BW-174	2014	Uganda	≥32	≥64	≥64	≥8	≥64	≥4	≥64	≤16	≥16	≤0.25	≤0.25	≥8	64	≥320
BW-175	2014	South Sudan	≥32	4	≥64	≥8	16	1	≤1	≤16	≥16	≤0.25	≤0.25	2	≤4	≥320
BW-176	2014	South Sudan	16	16	≥64	≥8	≤1	≤0.25	≤1	≤16	≥16	≤0.25	≤0.25	0.5	16	≤20
BW-177	2014	South Sudan	16	16	≤64	≥8		≤0.25	2	≤16	≥16	≤0.25	≤0.25	0.5	≤4	≥320
BW-178	2014	South Sudan	≥32	16	≥64	≥8	16	≤0.25	4	≤16	≤1	≤0.25	≤0.25	0.5	64	≥320
BW-179	2014	Uganda	≥32	≥64	≥64	≥8	16	≤0.25	32	≤16	≤1	≤0.25	≤0.25	1	24	≤20
BW-180	2013	South Sudan	≤2	≤1	≥64	≥8	≤1	1	≤1	≤16	≤1	≤0.25	≤0.25	4	≤4	≤20
BW-181	2014	South Sudan	16	4	≥64	≥8	16	≤0.25	4	≤16	≤1	≤0.25	≤0.26	≤0.25	≤4	≤20
BW-182	2014	Nigeria	≥32	16	≥64	≥8	4	1	2	≤16	≤1	≤0.25	≤0.25	1	≤4	≥320
BW-183	2014	Democratic Republic of the Congo	≥32	≥64	≥64	≥8	≥64	≥4	≥64	≤16	≥16	≤0.25	≤0.25	≥8	8	≥320
BW-184	2014	Mali	≤2	16	≥64	≥8	4	≤0.25	2	≤16	≤1	≤0.25	≤0.25	≤0.25	≤4	≥321
BW-185	2014	Mali	≥32	16	≥64	≥8	4	≤0.25	4	≤16	≤1	≤0.25	≤0.25	≤0.25	≤4	≥320
BW-186	2014	South Sudan	≥32	≥64	≥64	≥8	64	≥4	4	≤16	≥16	≤0.25	≤0.25	≥8	8	≥320
BW-187	2015	Mali	≤2	16	≥64	≥8	4	≤0.25	2	≤16	≤1	≤0.25	≤0.25	≤0.25	≤4	≥320
BW-188	2015	Not further defined African destinations	≥32	4	8	≥8	2	≤0.25	≤1	≤16	≤1	≤0.25	≤0.25	0.5	≤4	≥320
BW-189	2014	Unknown or multiple deployment settings	≥32	16	≥64	≥8	16	1	2	64	≥16	≤0.25	≤0.25	2	24	≥320
BW-192	2016	Sudan and Uganda	≥32	2	≥64	≥8	4	≤0.25	≤1	≤16	≤1	≤0.25	≤0.25	1	16	≥320
BW-193	2016	Nigeria	16	2	≥64	≥8	≤1	≥4	2	≤16	≥16	≤0.25	≤0.25	≥8	≤4	≥320
BW-194	2016	Mali	8	16	≥64	≥8	16	≥4	2	≤16	≤1	≤0.25	≤0.25	≥8	≤4	≤20
BW-195	2016	Mali	≤2	≥64	≥64	≥8	4	≤0.25	8	≤16	≤1	≤0.25	≤0.25	0.5	≤4	≤20
BW-198	2016	Ethiopia	≥32	32	≤64	≥8	≥64	≥4	8	≤16	≥16	≤0.25	≤0.25	≥8	24	≥320
BW-199	2016	Mali	16	16	≥64	≥8	4	≥4	2	≤16	≤1	≤0.25	≤0.25	≥8	≤4	≥320
BW-201	2016	India and Nepal	16	4	≥64	≥8	4	≤0.25	≤1	≤16	≥16	≤0.25	≤0.25	≤0.25	≤4	≥320
BW-203	2016	Sudan and Uganda	16	2	≥64	≥8	≤1	≤0.25	≤1	≤16	≤1	≤0.25	≤0.25	1	≤4	≥320
BW-206	2016	Mali	8	16	≥64	≥8	16	≥4	≥64	≤16	≤1	≤0.25	≤0.25	≥8	≤4	≥320
BW-207	2016	Iraq	16	16	≥64	≥8	16	0.5	2	≤16	≥16	≤0.25	≤0.25	1	≤4	≥320
BW-208	2016	Mali	≥32	16	≥64	≥8	16	≥4	≥64	≤16	≤1	≤0.25	≤0.25	≥8	8	≥320
BW-209	2016	Iraq	4	16	≥64	≥8	4	1	4	≤16	≥16	≤0.25	≤0.25	4	≤4	≥320

ASU: ampicillin/sulbactam, AZT: aztreonam, CTM: trimethoprim/sulfamethoxazole, CTX: cefotaxime, CPD: cefpodoxime, CTZ: ceftazidime, CIP: ciprofloxacin, ERT: ertapenem, FEP: cefepime, FOF: fosfomycin, GEN: gentamicin, IMI: imipenem, MER: meropenem, MOX: moxifloxacin, TZP: piperacillin/tazobactam.
